# Notes and Comments

**Published:** 1893-11

**Authors:** 


### Notes and Comments.[1]

ANTISEPTIC DENTISTRY.—In the transactions of the Illinois State Dental Society we find a very practical paper upon "Antiseptic Dentistry," by Dr. Matthew Newkirk, of Chicago. He says, in part, "A septic agent is simply that which contains the germ, the seed, the spore, the reproductive cell, of a low form of life, that with conditions favorable to itself is inimical and destructive to the substance belonging to another and higher form of life. . . . I was

[1] The assistant editor solicits contributions for this department,—new methods, new remedies and formulas, or any short practical note which may prove of value to the practitioner or student. Address 1506 Arch Street, Philadelphia.

somewhat surprised a year ago to hear a venerable and highly respected member of the dental profession antagonize the modern ideas of disinfection in this wise. As I remember, he said, 'We drink at fountains from cups which have passed from mouth to mouth and have not been disinfected; we go to hotels and use forks which have been in the mouths of we know not whom; we sit in seats in railroad cars that may be covered with germs; we ride in crowded street-cars laden with the breaths of many occupants; we do all this with comparative immunity. Why, then, should we be so extremely careful beyond ordinary washing of our dental instruments?'

"I say I was surprised, because such an argument shows plainly that his thinking had never been thorough enough to go to the core of the thing. The surgeon may open a great abscess, he may perform a laparotomy, where his hands are bathed in septic matter, and no harm follow to him, but if there be anywhere a *broken* surface, even a pin scratch, he is in imminent danger. Woe to him if with point of infected knife or needle he touches his own blood; his life may pay the forfeit, or disease may scourge him from the crown of his head to the sole of his foot.

"A young man of eighteen years, brother of one of my patients, applied to a surgeon for a slight operation on his foot, and had the misfortune to be inoculated with an infected bistoury. Abscesses followed in the lungs and elsewhere, and after suffering for months and undergoing several surgical operations, with no end of anxiety on the part of his family, he died. And all this loss and grief was suffered and borne because a certain man was ignorant, or lazy, or careless, and failed to disinfect one little instrument. If he now appreciates the truth, one would think his peace of mind would be gone forever. An experience like this would cloud the sky of a lifetime.

"That which holds true of the general surgeon is applicable to the dentist. *He uses a greater number of instruments liable to be infected in close proximity to territory favorable to infection than any other man on earth.* Every instrument which enters a carious tooth is likely to be infected by one or more of the agents or products of decay. A smooth excavator may not be, or if it is, may be readily cleansed, but a bur, with its many grooves, is certain to be, and is not easily cleansed. One of the most, if not the most dangerous instrument for infection is the bur when allowed to slip from the cavity and make a punctured wound of the soft parts."

Many cases of infection by dental instruments could be collected, and it can be truthfully said that a large number of our profession are not to-day practising antiseptic dentistry. This carelessness in these days can be considered little less than criminal. We have been surprised to find dentists of some "standing" allowing their soiled instruments to accumulate upon their bracket or in the drawer of the cabinet, and horrified to see instruments taken from the same drawer for use without ever giving them a thorough washing.

This is a subject upon which one can afford to be unusualy enthusiastic. It is one of vital importance to us as dentists and to our clients. Think, too, of a man operating with his finger-nails dirty, without washing his hands thoroughly before attending to each patient, using soiled linen, or, in cleansing the teeth of a patient, using a tooth-brush that has been used in some other mouth, as many do, because they are too indifferent or selfish to use a new one, or of a man who uses a piece of rubber dam a second time, washed or unwashed, or who fails to keep his cuspidors clean and free from odor. If he is careless in reference to these matters of personal cleanliness, he surely will be in regard to his instruments. We leave it to the reader as to what sort of man he is; surely he is not worthy the name of dentist.

---

Crania of Native Hawaiians.—At a recent meeting of the Academy of Natural Sciences of Philadelphia, Professor C. N. Peirce presented and described an interesting collection of ancient skulls of native Hawaiians estimated to be four hundred years old. These crania were secured through the personal efforts of Dr. J. M. Whitney, of Honolulu, an early graduate of the Pennsylvania College of Dental Surgery, who went to considerable trouble to make the collection as valuable as possible. From a letter to Dr. E. C. Kirk, and a subsequent one to Dr. Peirce, the following history is obtained.

The common people of years ago in that country were buried in the sand of the sea-shore, which is often thrown up into hills of considerable size by the trade-winds. The chiefs and men of note were laid away in caves with great secrecy and care. Tradition places these caves as burial-places of great antiquity. Many of the caves are miles from any passable road, the simple path being over the roughest and stoniest of ground, making travel very difficult.

With the bodies had been found a variety of objects, notably a quantity of ava, a root from which is still prepared a mild toxicant. The collection includes two skulls from the sand graves. These are easily distinguished from the others by their bleached appearance and smaller size.

Remarks on the value of the collection and the ethnographic and anatomical peculiarities of the specimens were also made by Dr. Harrison Allen.

---

The Forms of Edentulous Jaws in the Human Subject.—In a paper before the Academy of Natural Sciences of Philadelphia, Dr. Harrison Allen endeavors to demonstrate the peculiarities of the edentulous jaws of the human subject. This is of such peculiar interest to us as dentists that I give considerable space to the subject here. Among other things, Dr. Allen says it is rare to find an edentulous arch uniformly hyperostosed or uniformly atrophied, but they exhibit indications of changes different in character from the mere loss of the alveolar process.

The lower jaw passes up in front of the upper jaw in aged individuals who have lost teeth. As a result, the doctor claimed, the attrition of the incisorial region of the lower jaw is secured against the front of the upper jaw. The result attained by such attrition he called "shearing." Shearing takes place in proportion as the upper jaw at its anterior area is beaked.

Dr. Allen claims further that the loss of the alveolar process in the incisorial region of the upper jaw causes the incisive foramen to assume an absolutely new position in relation to the line of mastication. It exhibits a disposition to lie in the dental arch instead of back of it. The attrition by shearing, however, protects the contents of the foramen from pressure.

After giving tables of examination of a number of skulls, Dr. Allen says it is a noteworthy fact that from the entire series examined, only four showed complete absence of any secondary bone adaptation consequent upon the loss of the alveolar processes, and that all of these were from civilized races,—two ancient Egyptian and two Anglo-American.

The want of harmony between the secondary adaptations probably correlates with the irregular rate at which the teeth are lost. Individual peculiarities in this regard are doubtless numerous.

He also assumed that the coarse food of savage and semi-savage people caused the jaws, even in an edentulous condition, to be used actively in the act of mastication, while the more carefully prepared food suitable to the aged of civilized people enabled the jaws to have complete rest, and hence the mechanical conditions which predetermined the localization of new structures were not active.

The speaker concluded that the series of observations strengthens the position taken that the same forces which differentiate the kind of teeth operate in fashioning the shape of the jaws, even after the loss of the teeth.

Dr. C. N. Peirce, in alluding to Dr. Allen's paper, said that some of the prominences to which attention had been drawn were, in his estimation, due to the difference in the time of the loss of the teeth. Why some maxillaries of recent or present time should show complete atrophy, while others evidenced non-absorption or secondary development, he could not explain, but believed it was associated with temperamental and nutritional conditions. The development certainly indicated a healthy recuperative power on the part of the individual. With reference to the protrusion of the lower jaw and chin, and the change in adaptation of condyle to glenoid cavity, which Dr. Allen illustrated, Dr. Peirce thought they could be explained upon the principle of use and disuse, with adaptation of structures. In infancy the angle resulting from the relation of the ramus to the body of the bone was much greater than a right angle; indeed, the ramus was but little above the same horizontal plane occupied by the body of the bone, and the jaw was capable only of vertical and antero-posterior motion, such as is essential to sucking or nursing. As the three true or permanent molars are developed, the ramus assumes its vertical position, forming almost a right angle with the body of the bone, and at the same time making lateral or horizontal movement not only possible, but essential, this motion establishing the concomitant relation between the condyles and glenoid cavities. As these permanent molars later in life are lost, the force upon the jaw in occlusion is confined to the anterior part or incisive locality, which would necessarily tend to increase the angle and protrude the chin. This occurs sometimes quite early in life and while all the anterior teeth are in position. At the same time that the vertical motion is exerting this influence, the necessity for lateral motion has ceased by the loss of the grinders. This, as Dr. Peirce says, accounts for the change in the relative position of the condyle which was so well shown by the previous speaker, and which has been necessitated by a return to the vertical and antero-posterior motion common to infancy, with the loss of the horizontal or lateral motion of maturity.

